# Crystal structure of bis­(2-{1-[(*E*)-(4-fluoro­benz­yl)imino]­eth­yl}phenolato-κ^2^ *N*,*O*)palladium(II)

**DOI:** 10.1107/S2056989015004405

**Published:** 2015-03-11

**Authors:** Amalina Mohd Tajuddin, Hadariah Bahron, Hamizah Mohd Zaki, Karimah Kassim, Suchada Chantrapromma

**Affiliations:** a Faculty of Applied Sciences, Universiti Teknologi MARA, 40450 Shah Alam, Selangor, Malaysia; b DDH CoRe, Universiti Teknologi MARA, 40450 Shah Alam, Selangor, Malaysia; c Institute of Science, Universiti Teknologi MARA, 40450 Shah Alam, Selangor, Malaysia; d Department of Chemistry, Faculty of Science, Prince of Songkla University, Hat-Yai, Songkhla 90112, Thailand

**Keywords:** crystal structure, Pd^II^ complex, NO donors, Schiff base, catalyst activity, hydrogen bonding, π–π inter­actions

## Abstract

The asymmetric unit of the title complex, [Pd(C_15_H_13_FNO)_2_], contains one half of the mol­ecule with the Pd^II^ cation lying on an inversion centre and is coordinated by the bidentate Schiff base anion. The geometry around the cationic Pd^II^ centre is distorted square planar, chelated by the imine N- and phenolate O-donor atoms of the two Schiff base ligands. The N- and O-donor atoms of the two ligands are mutually *trans*, with Pd—N and Pd—O bond lengths of 2.028 (2) and 1.9770 (18) Å, respectively. The fluoro­phenyl ring is tilted away from the coordination plane and makes a dihedral angle of 66.2 (2)° with the phenolate ring. In the crystal, mol­ecules are linked into chains along the [101] direction by weak C—H⋯O hydrogen bonds. Weak π–π inter­actions with centroid–centroid distances of 4.079 (2) Å stack the mol­ecules along *c*.

## Abstract 2

In the square planar [Pd(C_15_H_13_FNO)_2_] complex, weak C—H⋯O and π–π inter­actions play important roles in the mol­ecular self-assembly, resulting in the formation of two-dimensional mol­ecular sheets which are further stacked along the *b* axis.

## Chemical context   

**Figure 1:**
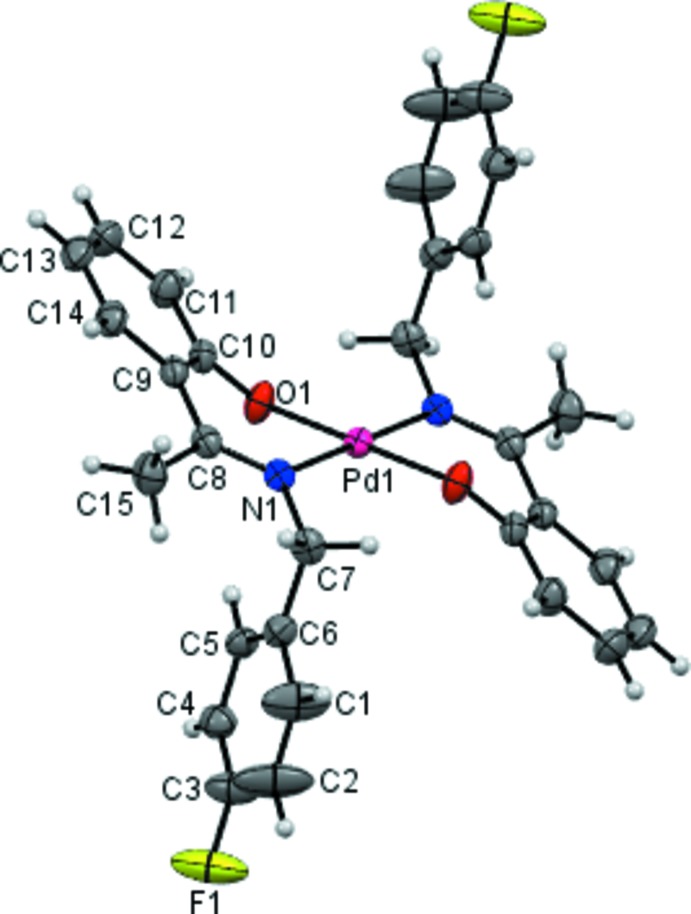
The mol­ecular structure of (1), showing 50% probability displacement ellipsoids and the atom-numbering scheme. The labelled atoms are related to the unlabelled atoms of the Schiff base ligands by the symmetry code 1 − *x*, 2 − *y*, 2 − *z*.

Schiff bases represent one of the most widely utilized classes of ligands in coordination chemistry and the chemistry of Schiff bases is still an area of increasing inter­est (Canali & Sherrigton, 1999 ▸). The Pd^II^ and Ni^II^ complexes of Schiff bases have attracted much attention as they play important roles in bioinorganic chemistry and may provide the basis for models of active sites of biological systems (Malik *et al.*, 2013 ▸) or act as catalysts (Shahnaz *et al.*, 2013 ▸). The title compound, [Pd(C_15_H_13_FNO)_2_], is related to the previously reported compound bis­{2-[(*E*)-(4-fluoro­benz­yl)imino­meth­yl]phen­o­lato-κ^2^ *N,O* ^1^}nickel(II) (Mohd Tajuddin *et al.*, 2014 ▸) in terms of the coordination geometry around the central metal. In this article, we report the synthesis of the title Schiff base–Pd^II^ complex and its characterization by spectroscopy and elemental analysis. The X-ray structure (Fig. 1 ▸), confirms the binding mode of the 4-fluoro­benz­yl(imino­eth­yl)phenolate ligand to the Pd^II^ cation.
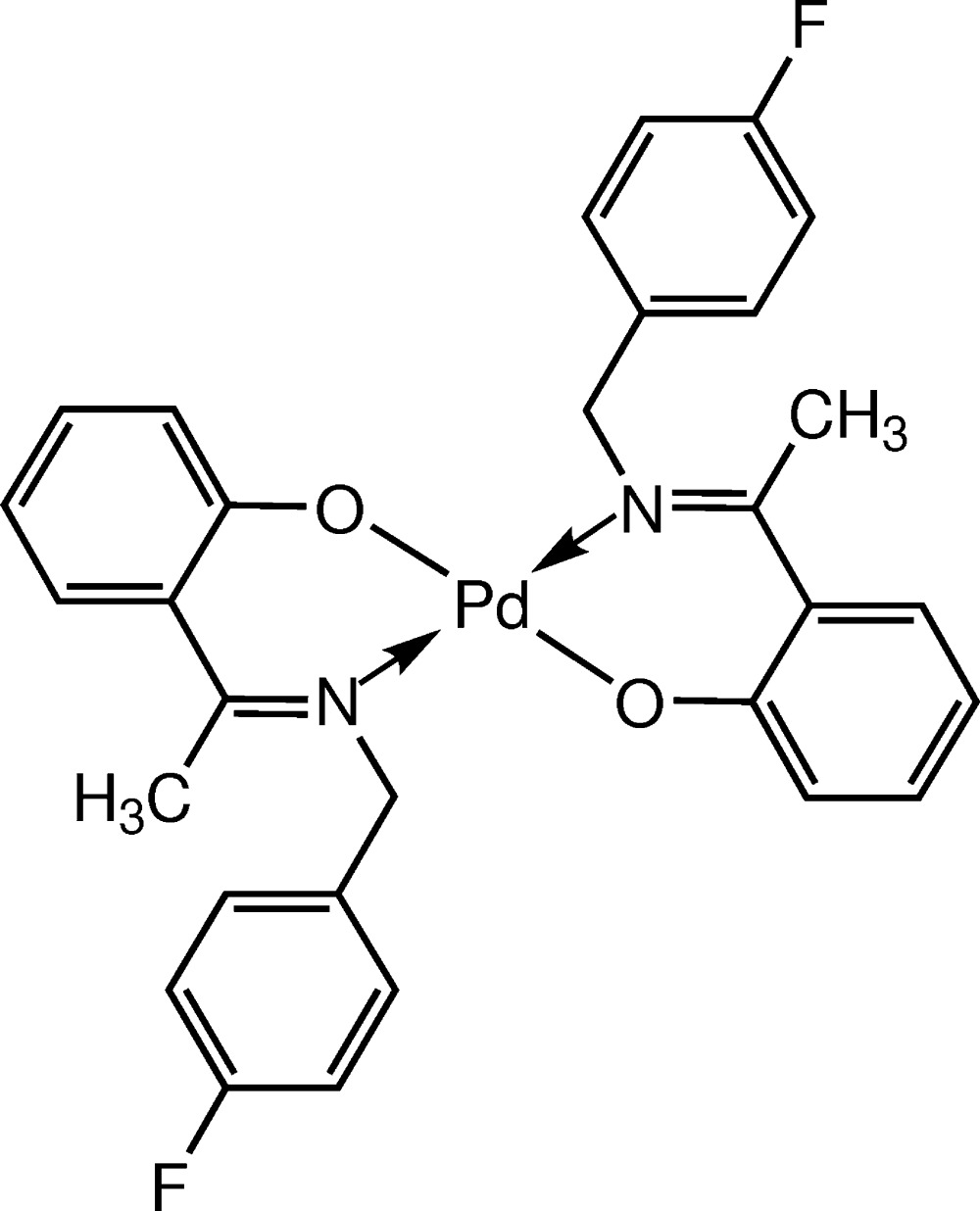


The title compound (1) was screened for catalytic activity in the Suzuki cross-coupling reaction between phenyl­boronic acid and iodo­benzene with a catalyst loading of 1 mmol%. The conversion of iodo­benzene was found to occur with a yield of 52%.

## Structural commentary   

The asymmetric unit of (1) contains one-half of the mol­ecule with the Pd^II^ cation lying on an inversion centre and the Schiff base anion acting as an *N*,*O* bidentate chelate ligand (Fig. 1 ▸). The Pd^II^ cation binds to the N and the O atoms of two symmetry-related Schiff base ligand such that the N and O atoms are mutually *trans*. The N_2_O_2_ donor sets of the two chelating Schiff base ligands in the equatorial plane around Pd1 adopt a slightly distorted square-planar coordination geometry. The Pd1—N1 and Pd1—O1 distances (Table 1 ▸) are typical of square-planar Pd^II^ complexes, and compare well with those observed in other closely related Pd^II^ complexes (Adrian *et al.*, 2008 ▸; Bahron *et al.*, 2014 ▸; Wan Ibrahim & Shamsuddin, 2012 ▸). The bite angle of the imino­methyl­phenolate chelate, N1—Pd1—O1 is 88.48 (8)°, which is also similar to that in a related Pd^II^ complex (Bahron *et al.*, 2014 ▸). The ring Pd1/N1/C8/C9/C10/O1 adopts an envelope conformation with Pd1 displaced by 0.270 (2) Å from the plane of the other ring atoms, and with puckering parameters *Q* = 0.525 (2) Å, θ = 112.8 (3) and ϕ = 206.9 (3)°. Other bond lengths and angles observed in the structure are also normal. The fluoro­phenyl ring (C1–C6) makes a dihedral angle of 66.2 (2)° with the phenolate ring (C9–C14).

## Supra­molecular features   

**Figure 2:**
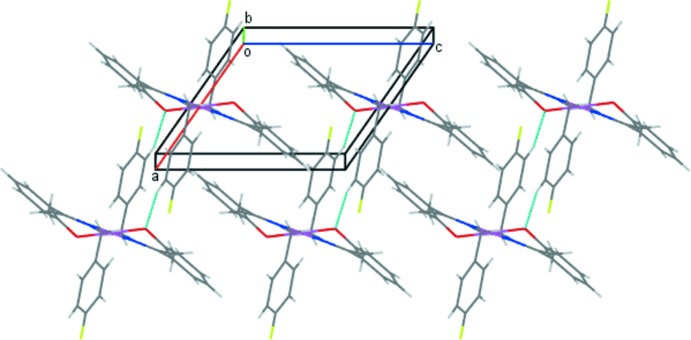
Screw chains of mol­ecules of (1) linked by C—H⋯O inter­actions (drawn as dashed lines).

**Figure 3:**
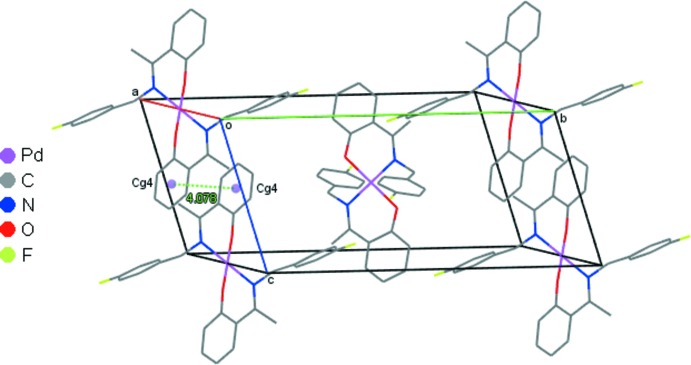
π–π contacts for (1) drawn as dotted lines with ring centroids shown as coloured spheres. *Cg*4 is the centroid of the C9–C14 ring. H atoms are omitted for clarity.

**Figure 4:**
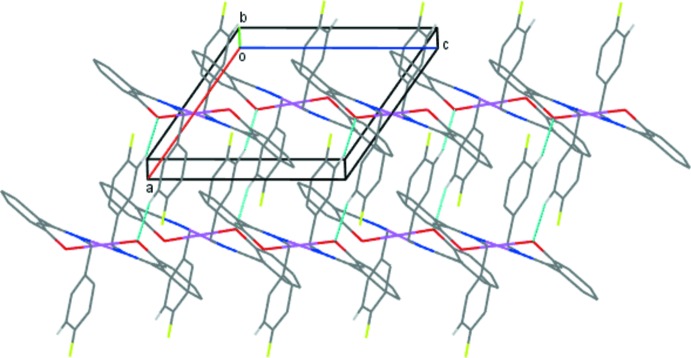
The packing of (1) viewed approximately along the *b* axis showing mol­ecular sheets of the Pd^II^ complex. Only H atoms involved in C—H⋯O inter­actions are shown for clarity.

In the crystal packing of (1), the mol­ecules are linked into chains along the [101] direction by weak C4—H4*A*⋯O1 inter­actions (Fig. 2 ▸, Table 2 ▸). A weak π–π stacking inter­action occurs between the phenolate rings of adjacent complexes (Fig. 3 ▸), with a centroid–centroid distance, *Cg*4⋯*Cg*4^iii^, of 4.079 (2) Å [symmetry code: (iii) = 1 − *x*, 2 − *y*, 1 − *z*; *Cg*4 is the centroid of the C9–C14 ring]. These combine with the C—H⋯O contacts to generate sheets in the *ac* plane (Fig. 4 ▸). These sheets are further stacked along the *b-*axis direction.

## Database survey   

Six Pd^II^ complexes with related Schiff base N_2_O_2_ donor sets have been reported (Brunner *et al.*, 2000 ▸; Mehta & Vengurlekar, 2001 ▸; Bahron *et al.*, 2011 ▸, 2014 ▸; Mohd Tajuddin *et al.*, 2012*a* ▸; Tsuno *et al.*, 2013 ▸). However, only three of these Pd^II^ complexes have closely related Schiff base ligands (Bahron *et al.*, 2011 ▸; 2014 ▸; Mohd Tajuddin *et al.*, 2012*a* ▸).

## Synthesis and crystallization   

The ligand, (*E*)-2-(1-(4-fluoro­benzyl­imino)­eth­yl)phenol (Mohd Tajuddin *et al.*, 2012*b* ▸) (2 mmol, 0.4877 g) was dissolved in CH_3_CN (30 mL) in a round-bottomed flask. Palladium(II) acetate (1 mmol, 0.2251 g) was dissolved separ­ately in CH_3_CN (20 mL) and was then added into the flask containing the ligand solution. The mixture was stirred and refluxed for 5 h upon which a turmeric yellow solid was formed. The solid was filtered off, washed with ice-cold CH_3_CN and air dried at room temperature. The solid product was recrystallized from chloro­form, yielding yellow crystals (yield 48.5%). ^1^H NMR, ^13^C NMR and IR spectral bands have been studied and agree well with the structure obtained from the values of the CHN analyses and X-ray structure determination.

Melting point 508–510 K. Analytical data for C_30_H_26_F_2_N_2_O_2_Pd: C, 60.97; H, 4.43; N, 4.74%; Found: C, 60.81; H, 4.49; N, 4.66%. IR (KBr, cm^−1^): 1598 ν(C=N), 1319 ν(C—N), 1216 ν(C—O), 1321 ν(CH_3_), 556 ν(Pd—N), 450 ν(Pd—O). ^1^H NMR (300 MHz, CDCl_3_): δ (p.p.m.) 2.32 (*s*, 3H, C—CH_3_), 5.11 (*s*, 2H, CH_2_), 6.53–7.46 (ArC). ^13^C NMR (300 MHz, CDCl_3_): δ (p.p.m.) 19.5 (C—CH_3_), 54.2 (CH_2_), 115.3, 115.6, 121.3, 128.6, 130.2 (ArC), 169.8 (C=N).

The infrared spectrum of (1) exhibits a strong band at 1598 cm^−1^ assignable to the C=N stretching frequency of the azomethine moiety. Weak bands at 556 and 450 cm^−1^ attributable to Pd—N and Pd—O vibrations, respectively (Ouf *et al.*, 2010 ▸), are due to the participation of the nitro­gen of the azomethine group and the oxygen of the phenolate ring in the complexation of the palladium(II) centre by the Schiff base ligands. From the NMR results, the free 4-fluoro­benz­yl(imino­eth­yl)phenolate ligand shows a multiplet at around 6.80–7.57 p.p.m. assignable to the aromatic protons. A corres­ponding multiplet appears in almost the same position in the spectrum of the Pd^II^ complex (compound 1) as that observed by Gupta *et al.* (2013 ▸). Singlets for aliphatic methyl­ene (–CH_2_) and methyl (–CH_3_) protons appear at 5.11 and 2.32 p.p.m., respectively. The ^13^C chemical shift for the imine carbon (C=N) is found at 169.8 p.p.m., agreeing with data reported by Şenol *et al.* (2011 ▸).

The title compound was screened for catalytic activity in the Suzuki cross-coupling reaction between phenyl­boronic acid with iodo­benzene. The reaction was carried out under nitro­gen at 373 K in di­methyl­acetamide with a catalyst loading of 1 mmol%. The conversion of iodo­benzene was monitored using GC–FID after 24 hours of reaction time. This resulted in a 52% conversion of iodo­benzene in the reaction.

## Refinement   

Crystal data, data collection and crystal structure refinement details are summarized in Table 3 ▸. All H atoms were positioned geometrically and allowed to ride on their parent atoms, with *d*(C—H) = 0.93 Å for aromatic, 0.97 Å for CH_2_ and 0.96 Å for CH_3_ hydrogen atoms. The *U*_iso_ values were constrained to be 1.5*U*_eq_ of the carrier atom for methyl H atoms and 1.2*U*_eq_ for the remaining H atoms. A rotating group model was used for the methyl groups.

## Supplementary Material

Crystal structure: contains datablock(s) global, I. DOI: 10.1107/S2056989015004405/sj5444sup1.cif

Structure factors: contains datablock(s) I. DOI: 10.1107/S2056989015004405/sj5444Isup2.hkl

CCDC reference: 1045879

Additional supporting information:  crystallographic information; 3D view; checkCIF report

## Figures and Tables

**Table 1:** Selected geometric parameters (, )

Pd1O1	1.9770(18)	Pd1N1	2.028(2)
			
O1Pd1N1	88.48(8)	O1^i^Pd1N1	91.52(8)

Symmetry code: (i) 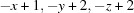.

**Table 2:** Hydrogen-bond geometry (, )

*D*H*A*	*D*H	H*A*	*D* *A*	*D*H*A*
C4H4*A*O1^ii^	0.93	2.50	3.405(5)	165

Symmetry code: (ii) 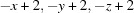.

**Table 3:** Experimental details

Crystal data	
Chemical formula	[Pd(C_15_H_13_FNO)_2_]
*M*_r_	590.93
Crystal system, space group	Monoclinic, *P*2_1_/*c*
Temperature (K)	296
*a*, *b*, *c* ()	7.5924(5), 21.9212(14), 9.3475(5)
()	124.963(4)
*V* (^3^)	1274.97(15)
*Z*	2
Radiation type	Mo *K*
(mm^1^)	0.77
Crystal size (mm)	0.50 0.25 0.25

Data collection	
Diffractometer	Bruker APEXII CCD area detector
Absorption correction	Multi-scan (*SADABS*; Bruker, 2009 ▸)
*T*_min_, *T*_max_	0.699, 0.830
No. of measured, independent and observed [*I* > 2(*I*)] reflections	38866, 2776, 2720
*R*_int_	0.057
(sin /)_max_ (^1^)	0.639

Refinement	
*R*[*F* ^2^ > 2(*F* ^2^)], *wR*(*F* ^2^), *S*	0.034, 0.073, 1.30
No. of reflections	2776
No. of parameters	170
H-atom treatment	H-atom parameters constrained
_max_, _min_ (e ^3^)	0.24, 0.48

Computer programs: *APEX2* and *SAINT* (Bruker, 2009 ▸), *SHELXTL* (Sheldrick, 2008 ▸), *PLATON* (Spek, 2009 ▸), *Mercury* (Macrae *et al.*, 2006 ▸) and *publCIF* (Westrip, 2010 ▸).

## supplementary crystallographic information

### Crystal data

**Table:**

[Pd(C_15_H_13_FNO)_2_]	*F*(000) = 600
*M**_r_* = 590.93	*D*_x_ = 1.539 Mg m^−^^3^
Monoclinic, *P*2_1_/*c*	Melting point = 508–510 K
Hall symbol: -P 2ybc	Mo *K*α radiation, λ = 0.71073 Å
*a* = 7.5924 (5) Å	Cell parameters from 2776 reflections
*b* = 21.9212 (14) Å	θ = 3.2–27.0°
*c* = 9.3475 (5) Å	µ = 0.77 mm^−^^1^
β = 124.963 (4)°	*T* = 296 K
*V* = 1274.97 (15) Å^3^	Block, yellow
*Z* = 2	0.50 × 0.25 × 0.25 mm

### Data collection

**Table:**

Bruker APEXII CCD area-detector diffractometer	2776 independent reflections
Radiation source: sealed tube	2720 reflections with *I* > 2σ(*I*)
Graphite monochromator	*R*_int_ = 0.057
φ and ω scans	θ_max_ = 27.0°, θ_min_ = 3.2°
Absorption correction: multi-scan (*SADABS*; Bruker, 2009)	*h* = −9→9
*T*_min_ = 0.699, *T*_max_ = 0.830	*k* = −28→28
38866 measured reflections	*l* = −11→11

### Refinement

**Table:**

Refinement on *F*^2^	Primary atom site location: structure-invariant direct methods
Least-squares matrix: full	Secondary atom site location: difference Fourier map
*R*[*F*^2^ > 2σ(*F*^2^)] = 0.034	Hydrogen site location: inferred from neighbouring sites
*wR*(*F*^2^) = 0.073	H-atom parameters constrained
*S* = 1.30	*w* = 1/[σ^2^(*F*_o_^2^) + (0.0086*P*)^2^ + 1.3994*P*] where *P* = (*F*_o_^2^ + 2*F*_c_^2^)/3
2776 reflections	(Δ/σ)_max_ < 0.001
170 parameters	Δρ_max_ = 0.24 e Å^−^^3^
0 restraints	Δρ_min_ = −0.48 e Å^−^^3^

### Special details

**Table:**

Geometry. All esds (except the esd in the dihedral angle between two l.s. planes) are estimated using the full covariance matrix. The cell esds are taken into account individually in the estimation of esds in distances, angles and torsion angles; correlations between esds in cell parameters are only used when they are defined by crystal symmetry. An approximate (isotropic) treatment of cell esds is used for estimating esds involving l.s. planes.
Refinement. Refinement of F^2^ against ALL reflections. The weighted R-factor wR and goodness of fit S are based on F^2^, conventional R-factors R are based on F, with F set to zero for negative F^2^. The threshold expression of F^2^ > 2sigma(F^2^) is used only for calculating R-factors(gt) etc. and is not relevant to the choice of reflections for refinement. R-factors based on F^2^ are statistically about twice as large as those based on F, and R- factors based on ALL data will be even larger.

### Fractional atomic coordinates and isotropic or equivalent isotropic displacement parameters (Å^2^)

**Table:**

	*x*	*y*	*z*	*U*_iso_*/*U*_eq_	
Pd1	0.5000	1.0000	1.0000	0.03202 (9)	
F1	1.3139 (4)	0.77987 (11)	1.2186 (5)	0.1153 (11)	
O1	0.5384 (3)	1.04581 (9)	0.8375 (2)	0.0462 (5)	
N1	0.4268 (3)	0.92521 (9)	0.8478 (3)	0.0343 (4)	
C1	0.7655 (7)	0.78728 (16)	1.0865 (7)	0.0924 (16)	
H1A	0.6670	0.7629	1.0890	0.111*	
C2	0.9701 (7)	0.76567 (18)	1.1567 (8)	0.117 (2)	
H2A	1.0108	0.7272	1.2078	0.140*	
C3	1.1113 (6)	0.80149 (15)	1.1502 (6)	0.0716 (11)	
C4	1.0618 (5)	0.85845 (14)	1.0834 (4)	0.0495 (7)	
H4A	1.1627	0.8827	1.0839	0.059*	
C5	0.8555 (5)	0.87966 (12)	1.0140 (4)	0.0416 (6)	
H5	0.8184	0.9188	0.9670	0.050*	
C6	0.7048 (5)	0.84467 (12)	1.0125 (4)	0.0411 (6)	
C7	0.4795 (5)	0.86633 (12)	0.9399 (4)	0.0429 (6)	
H7A	0.3781	0.8358	0.8598	0.051*	
H7B	0.4629	0.8702	1.0349	0.051*	
C8	0.3497 (4)	0.92650 (12)	0.6831 (4)	0.0382 (6)	
C9	0.2963 (4)	0.98338 (13)	0.5855 (3)	0.0381 (6)	
C10	0.3929 (4)	1.03958 (13)	0.6678 (3)	0.0390 (6)	
C11	0.3352 (6)	1.09165 (15)	0.5625 (4)	0.0527 (7)	
H11A	0.3971	1.1289	0.6147	0.063*	
C12	0.1898 (6)	1.08905 (16)	0.3845 (4)	0.0578 (8)	
H12A	0.1554	1.1243	0.3181	0.069*	
C13	0.0945 (5)	1.03431 (17)	0.3037 (4)	0.0551 (8)	
H13A	−0.0052	1.0326	0.1833	0.066*	
C14	0.1479 (5)	0.98286 (15)	0.4023 (4)	0.0482 (7)	
H14A	0.0844	0.9461	0.3468	0.058*	
C15	0.3123 (6)	0.86788 (14)	0.5833 (4)	0.0566 (8)	
H15A	0.3280	0.8756	0.4900	0.085*	
H15B	0.1698	0.8531	0.5359	0.085*	
H15C	0.4154	0.8378	0.6608	0.085*	

### Atomic displacement parameters (Å^2^)

**Table:**

	*U*^11^	*U*^22^	*U*^33^	*U*^12^	*U*^13^	*U*^23^
Pd1	0.03017 (15)	0.03263 (14)	0.03135 (14)	−0.00387 (10)	0.01650 (11)	−0.00210 (11)
F1	0.0547 (14)	0.0598 (14)	0.175 (3)	0.0194 (11)	0.0329 (16)	0.0054 (16)
O1	0.0568 (12)	0.0479 (11)	0.0349 (10)	−0.0199 (9)	0.0269 (9)	−0.0048 (8)
N1	0.0332 (11)	0.0332 (10)	0.0370 (11)	−0.0041 (8)	0.0203 (9)	−0.0044 (9)
C1	0.062 (2)	0.0424 (19)	0.143 (4)	−0.0064 (17)	0.041 (3)	0.027 (2)
C2	0.066 (3)	0.042 (2)	0.183 (6)	0.0083 (19)	0.036 (3)	0.038 (3)
C3	0.0486 (19)	0.0420 (17)	0.089 (3)	0.0076 (15)	0.0185 (19)	−0.0048 (17)
C4	0.0444 (16)	0.0509 (17)	0.0489 (17)	0.0005 (13)	0.0242 (14)	−0.0016 (13)
C5	0.0495 (16)	0.0367 (13)	0.0406 (14)	0.0042 (12)	0.0269 (13)	0.0054 (11)
C6	0.0476 (15)	0.0316 (12)	0.0381 (14)	−0.0029 (11)	0.0211 (12)	−0.0027 (11)
C7	0.0504 (16)	0.0328 (13)	0.0514 (16)	−0.0084 (11)	0.0327 (14)	−0.0041 (12)
C8	0.0335 (13)	0.0426 (14)	0.0395 (14)	−0.0059 (11)	0.0215 (12)	−0.0100 (11)
C9	0.0350 (13)	0.0478 (15)	0.0337 (13)	−0.0014 (11)	0.0209 (11)	−0.0032 (11)
C10	0.0424 (14)	0.0460 (15)	0.0363 (14)	−0.0025 (12)	0.0271 (12)	−0.0016 (11)
C11	0.067 (2)	0.0483 (17)	0.0498 (18)	−0.0012 (15)	0.0373 (17)	0.0029 (13)
C12	0.065 (2)	0.062 (2)	0.0513 (18)	0.0141 (16)	0.0365 (17)	0.0169 (16)
C13	0.0447 (17)	0.079 (2)	0.0360 (15)	0.0042 (16)	0.0197 (13)	0.0037 (15)
C14	0.0422 (15)	0.0614 (18)	0.0375 (15)	−0.0046 (13)	0.0208 (13)	−0.0066 (13)
C15	0.070 (2)	0.0485 (17)	0.0523 (18)	−0.0102 (15)	0.0360 (17)	−0.0160 (14)

### Geometric parameters (Å, º)

**Table:**

Pd1—O1	1.9770 (18)	C6—C7	1.510 (4)
Pd1—O1^i^	1.9770 (18)	C7—H7A	0.9700
Pd1—N1^i^	2.028 (2)	C7—H7B	0.9700
Pd1—N1	2.028 (2)	C8—C9	1.458 (4)
F1—C3	1.369 (4)	C8—C15	1.516 (4)
O1—C10	1.321 (3)	C9—C14	1.411 (4)
N1—C8	1.297 (3)	C9—C10	1.415 (4)
N1—C7	1.474 (3)	C10—C11	1.403 (4)
C1—C2	1.378 (6)	C11—C12	1.373 (5)
C1—C6	1.381 (4)	C11—H11A	0.9300
C1—H1A	0.9300	C12—C13	1.381 (5)
C2—C3	1.358 (6)	C12—H12A	0.9300
C2—H2A	0.9300	C13—C14	1.363 (5)
C3—C4	1.349 (5)	C13—H13A	0.9300
C4—C5	1.387 (4)	C14—H14A	0.9300
C4—H4A	0.9300	C15—H15A	0.9600
C5—C6	1.371 (4)	C15—H15B	0.9600
C5—H5	0.9300	C15—H15C	0.9600
			
O1—Pd1—O1^i^	180.00 (10)	N1—C7—H7B	108.9
O1—Pd1—N1^i^	91.52 (8)	C6—C7—H7B	108.9
O1^i^—Pd1—N1^i^	88.48 (8)	H7A—C7—H7B	107.7
O1—Pd1—N1	88.48 (8)	N1—C8—C9	122.4 (2)
O1^i^—Pd1—N1	91.52 (8)	N1—C8—C15	120.7 (3)
N1^i^—Pd1—N1	180.000 (1)	C9—C8—C15	116.9 (2)
C10—O1—Pd1	118.68 (16)	C14—C9—C10	118.1 (3)
C8—N1—C7	120.0 (2)	C14—C9—C8	119.6 (3)
C8—N1—Pd1	124.82 (18)	C10—C9—C8	122.2 (2)
C7—N1—Pd1	115.13 (17)	O1—C10—C11	117.9 (3)
C2—C1—C6	120.9 (4)	O1—C10—C9	124.0 (2)
C2—C1—H1A	119.6	C11—C10—C9	118.1 (3)
C6—C1—H1A	119.6	C12—C11—C10	121.8 (3)
C3—C2—C1	118.9 (4)	C12—C11—H11A	119.1
C3—C2—H2A	120.6	C10—C11—H11A	119.1
C1—C2—H2A	120.6	C11—C12—C13	120.3 (3)
C4—C3—C2	122.6 (3)	C11—C12—H12A	119.9
C4—C3—F1	118.5 (4)	C13—C12—H12A	119.9
C2—C3—F1	118.9 (3)	C14—C13—C12	119.3 (3)
C3—C4—C5	117.8 (3)	C14—C13—H13A	120.3
C3—C4—H4A	121.1	C12—C13—H13A	120.3
C5—C4—H4A	121.1	C13—C14—C9	122.4 (3)
C6—C5—C4	121.9 (3)	C13—C14—H14A	118.8
C6—C5—H5	119.0	C9—C14—H14A	118.8
C4—C5—H5	119.0	C8—C15—H15A	109.5
C5—C6—C1	117.9 (3)	C8—C15—H15B	109.5
C5—C6—C7	123.5 (2)	H15A—C15—H15B	109.5
C1—C6—C7	118.6 (3)	C8—C15—H15C	109.5
N1—C7—C6	113.4 (2)	H15A—C15—H15C	109.5
N1—C7—H7A	108.9	H15B—C15—H15C	109.5
C6—C7—H7A	108.9		
			
N1^i^—Pd1—O1—C10	−134.7 (2)	Pd1—N1—C8—C9	−3.2 (4)
N1—Pd1—O1—C10	45.3 (2)	C7—N1—C8—C15	0.2 (4)
O1—Pd1—N1—C8	−25.1 (2)	Pd1—N1—C8—C15	176.5 (2)
O1^i^—Pd1—N1—C8	154.9 (2)	N1—C8—C9—C14	−157.8 (3)
O1—Pd1—N1—C7	151.39 (18)	C15—C8—C9—C14	22.6 (4)
O1^i^—Pd1—N1—C7	−28.61 (18)	N1—C8—C9—C10	24.0 (4)
C6—C1—C2—C3	−0.8 (9)	C15—C8—C9—C10	−155.6 (3)
C1—C2—C3—C4	2.6 (9)	Pd1—O1—C10—C11	141.1 (2)
C1—C2—C3—F1	−179.5 (5)	Pd1—O1—C10—C9	−40.7 (3)
C2—C3—C4—C5	−2.3 (7)	C14—C9—C10—O1	−178.0 (3)
F1—C3—C4—C5	179.8 (3)	C8—C9—C10—O1	0.2 (4)
C3—C4—C5—C6	0.2 (5)	C14—C9—C10—C11	0.2 (4)
C4—C5—C6—C1	1.4 (5)	C8—C9—C10—C11	178.4 (3)
C4—C5—C6—C7	179.4 (3)	O1—C10—C11—C12	178.2 (3)
C2—C1—C6—C5	−1.1 (7)	C9—C10—C11—C12	−0.2 (5)
C2—C1—C6—C7	−179.1 (5)	C10—C11—C12—C13	0.4 (5)
C8—N1—C7—C6	87.6 (3)	C11—C12—C13—C14	−0.7 (5)
Pd1—N1—C7—C6	−89.0 (2)	C12—C13—C14—C9	0.8 (5)
C5—C6—C7—N1	8.3 (4)	C10—C9—C14—C13	−0.6 (4)
C1—C6—C7—N1	−173.7 (3)	C8—C9—C14—C13	−178.8 (3)
C7—N1—C8—C9	−179.5 (2)		

Symmetry code: (i) −*x*+1, −*y*+2, −*z*+2.

### Hydrogen-bond geometry (Å, º)

**Table:**

*D*—H···*A*	*D*—H	H···*A*	*D*···*A*	*D*—H···*A*
C4—H4*A*···O1^ii^	0.93	2.50	3.405 (5)	165

Symmetry code: (ii) −*x*+2, −*y*+2, −*z*+2.
